# Reliability of dynamic contrast-enhanced magnetic resonance imaging data in primary brain tumours: a comparison of Tofts and shutter speed models

**DOI:** 10.1007/s00234-019-02265-2

**Published:** 2019-08-07

**Authors:** Marianna Inglese, Katherine L. Ordidge, Lesley Honeyfield, Tara D. Barwick, Eric O. Aboagye, Adam D. Waldman, Matthew Grech-Sollars

**Affiliations:** 1 Department of Surgery and Cancer, GN1 Commonwealth building, Hammersmith Hospital, Imperial College London, Du Cane Road, London, W12 0NN UK; 2 Department of Imaging, Imperial College Healthcare NHS Trust, London, UK; 3 Department of Medicine, Imperial College London, London, UK; 4 Centre for Clinical Brain Sciences, The University of Edinburgh, Edinburgh, UK

**Keywords:** Glioma, DCE-MRI, Shutter speed model, Tofts model, Primary brain tumour

## Abstract

**Purpose:**

The purpose of this study is to investigate the robustness of pharmacokinetic modelling of DCE-MRI brain tumour data and to ascertain reliable perfusion parameters through a model selection process and a stability test.

**Methods:**

DCE-MRI data of 14 patients with primary brain tumours were analysed using the Tofts model (TM), the extended Tofts model (ETM), the shutter speed model (SSM) and the extended shutter speed model (ESSM). A no-effect model (NEM) was implemented to assess overfitting of data by the other models. For each lesion, the Akaike Information Criteria (AIC) was used to build a 3D model selection map. The variability of each pharmacokinetic parameter extracted from this map was assessed with a noise propagation procedure, resulting in voxel-wise distributions of the coefficient of variation (CV).

**Results:**

The model selection map over all patients showed NEM had the best fit in 35.5% of voxels, followed by ETM (32%), TM (28.2%), SSM (4.3%) and ESSM (< 0.1%). In analysing the reliability of *K*^trans^, when considering regions with a CV < 20%, ≈ 25% of voxels were found to be stable across all patients. The remaining 75% of voxels were considered unreliable.

**Conclusions:**

The majority of studies quantifying DCE-MRI data in brain tumours only consider a single model and whole tumour statistics for the output parameters. Appropriate model selection, considering tissue biology and its effects on blood brain barrier permeability and exchange conditions, together with an analysis on the reliability and stability of the calculated parameters, is critical in processing robust brain tumour DCE-MRI data.

## Introduction

Dynamic contrast-enhanced magnetic resonance imaging (DCE-MRI) is a non-invasive methodology that allows tissue perfusion and permeability to be quantified through analysis of T_1_-weighted MR images acquired before, during and after an intravenous (IV) injection of a gadolinium-based contrast agent (CA).

Nowadays, DCE-MRI finds application in a wide range of oncological studies. In brain tumours, the Tofts model (TM), also known as the standard model, together with its extended version (EMT), are regularly applied [1–6]. There are a few examples of the application of shutter speed model (SSM) in brain tumours in the literature [7, 8] while it has been mainly implemented in the study of breast cancer [9], prostate cancer [10] and hepatocellular carcinoma [11]. The extended shutter speed model (ESSM), also called second generation shutter speed model, or BALDERO (blood agent level dependent and extravasation relaxation overview), has previously found applications only in hepatocellular carcinoma [12] and simulated data [13]. These four models assume that the CA passes readily between the intravascular compartment and the tissue interstitium. However, this assumption is not valid in the presence of the intact blood-brain barrier (BBB), where there is negligible leakage. In this case the CA will only affect the intravascular T_1_ value on first pass of the bolus. In addition, the vascular component of a brain tumour is heterogeneous and certain regions may not be perfused (e.g. necrotic regions). We therefore also implemented a no-effect model (NEM) which assumes negligible effect of the CA on the voxel T_1_ properties.

In the last decade, the number of pharmacokinetic studies of DCE-MRI brain data has increased as this quantification technique has been found to be indicative of the malignant grade of brain tumours [14]. However, two main issues need to be taken into account when analysing DCE-MRI data. Firstly, pharmacokinetic studies often show different and discordant results, thus bringing the reliability of this quantification technique into question [15, 16]. In fact, DCE-MRI data analysis is affected by (a) the acquisition protocol (trade-off between spatial and temporal resolution) [17] and (b) the quantification procedure. Furthermore, most studies present only results from application of a single pharmacokinetic model and the consequent statistical analysis of averaged values evaluated over the whole tumour volume [18–20]. This approach completely ignores the particularly heterogeneous nature of brain tumour vasculature and vascular permeability.

In this prospective study, we investigate the robustness of pharmacokinetic modelling of DCE-MRI brain data. We propose a method to identify reliable DCE-MRI data based on a model selection procedure, building on previous work by Bagher-Ebadian et al. [3], and a stability test: five different pharmacokinetic models (TM, ETM, SSM, ESSM and NEM) are assessed and the Akaike information criteria index is used for model selection; we then evaluate the stability of each parameter extracted from the model of choice, in terms of coefficients of variations (CVs), through a noise propagation procedure.

## Materials and methods

### Patient population

Fourteen patients (7 male, 7 female; aged 23–73 years, mean 40 years) with primary brain tumours were recruited to this study. Ethical approval was given by the local ethics committee and informed consent was obtained from all patients. Patients had an MRI at diagnosis, prior to receiving any treatment. Following surgery, histopathological data showed three patients with WHO grade IV glioblastoma, two patients with WHO grade III astrocytoma, two patients with WHO grade III oligodendroglioma, three patients with WHO grade II astrocytoma, three patients with WHO grade II oligodendroglioma and one patient with a WHO grade I dysembryoplastic neuroepithelial tumour.

### DCE-MRI data acquisition

MR images were acquired on a 3-T Siemens Verio MRI system using a 32 channel head coil; including pre- and post-contrast T_1_-weighted images, T_2_-FLAIR images and a DCE sequence with a variable flip angle pre-contrast T_1_ map acquisition. The DCE-MRI protocol included five pre-contrast spoiled gradient recalled echo (SPGRE) 3D vibe sequences at five different flip angles (2, 8, 12, 15, 20, 26), and a dynamic 3D vibe sequence (TR = 3.34 ms, TE = 0.99 ms, flip angle = 26, FOV 240 × 240 mm, acquisition matrix 128 × 128, slice thickness 5 mm, slice gap 1 mm, 80 volumes). To obtain acquisitions before, during and after the injection of the CA, 0.1 mmol/kg body-weight gadolinium-diethylene triaminepentacetate (Gd-DTPA, Gadovist) was injected using a power injector on the fifth acquisition using a flow rate of 3 ml/s immediately followed by 20 ml saline solution. The 3D acquisition allowed us to cover the entire brain; 80 time points were acquired with an average temporal resolution of 2.89 s and a total acquisition time of ≈ 4 min.

### DCE-MRI data analysis

Volumes of interest (VOIs) were drawn by a Radiologist and confirmed by a Consultant Neuroradiologist for each patient around T_2_-FLAIR hyperintense regions and on a 2-cm-diameter circular region in normal-appearing contra-lateral white matter. DCE-MRI data were analysed using a semi-automated in-house software written in MATLAB (Mathworks, R2017a). Before the application of any of the aforementioned pharmacokinetic models, we calculated the relaxation rate at baseline (R_10_) and relaxed signal (M_0_) as 3D maps, with the Ernst formula (assuming TE < <T_2_*) using the set of SPGRE pre-contrast images acquired at different flip angles [21]:

1 $$ S\left(\alpha \right)={M}_0\ \sin \left(\alpha \right)\frac{1-{e}^{-{R}_{10} TR}}{1-\cos \left(\alpha \right){e}^{-{R}_{10} TR}} $$

where α is the flip angle having values [(2, 8, 12, 15, 20, 26]) and TR is 3.34 ms. Reformulating Eq. 1 as a linear regression system (y = cx + d) following the method described by Liberman et al. in [22], gives:

2 $$ \frac{S\left(\alpha \right)}{\sin \left(\alpha \right)}=E\frac{S\left(\alpha \right)}{\tan \left(\alpha \right)}+{M}_0\left(1-E\right) $$

where $$ E={e}^{-{R}_{10} TR} $$.

The slope c = E and intercept d = M_0_ (1 − E) can thus be estimated and continuing from [22], R_10_ and M_0_ can then be obtained through:

3 $$ {R}_{10}=-\frac{\log (c)}{TR},\kern0.5em {M}_0=\frac{d}{1-c} $$

Then, 4D (x, y, z, t) post-injection longitudinal relaxation rate *R*_1_(*t*) maps for each dynamic phase are calculated using signal intensity data from the post-contrast dynamic series:

4 $$ {R}_1(t)=-\left(1/ TR\right)\log \frac{1-\left(A+B\right)}{1-\cos \left(\alpha \right)\left(A+B\right)} $$

where α = 26, A = S(*t*) − S(0)/M_0_ sin(*α*), B = (1 − E)/1 − E · cos(*α*). S(0) and S(t) are the pre-contrast injection signal intensity and the signal at the dynamic phase *t*, respectively [23].

The longitudinal relaxation rate is determined in order to calculate the concentration of the CA. This is done through a calibration between the concentration of CA [CA] and the measured H_2_O MR signal. This can be modelled by either a linear or nonlinear relationship as described below.

#### Tofts model (TM)

In applying the Tofts model to brain tumours, we expressed the flux of the tracer across two well-mixed compartments (blood and the extravascular, extracellular space) through the volume transfer constant K^trans^ [24]. The TM, also called the standard model, assumes negligible plasma compartment and can describe weakly vascularized brain tissue. More importantly, it assumes a linear dependence of R_1_ on [CA] (that is the equivalent of assuming the equilibrium transcytolemmal water exchange kinetics in the fast exchange limit(FXL)):

5 $$ {R}_1(t)={r}_1\left[ CA(t)\right]+{R}_{10} $$

where *r*_1_ is the CA relaxivity.

The extravasation of the contrast from the plasma to the extravascular extracellular space (EES) was accounted by the Kety-Schmidt rate law [25]:

6 $$ \left[{CA}_o(T)\right]={K}^{trans}{\int}_0^t\left[{CA}_p(t)\right]{e}^{\frac{K^{trans}\left(T-t\right)}{v_e}} dt $$

where *K*^trans^ is the first-order rate constant for plasma to interstitium CA transport (min^−1^) and *v*_e_ is a measure of the EES volume fraction.

The ratio between *K*^trans^ and *v*_e_ results in the third pharmacokinetic parameter *k*_ep_, which is the back flux rate constant (min^−1^). [CA_0_] and [CA_p_] are the concentration of CA in the ‘outside’ space (the extravascular extracellular space) and in the plasma, respectively. [CA_p_] is also called the arterial input function (AIF). The fitting of Eq. 6 was performed using the inbuilt MATLAB *fminsearch* function, which uses the Nelder-Mead simplex algorithm as described in Lagarias et al. [26]. The minimization procedure is done voxel-wise in order to obtain a 3D map for each pharmacokinetic parameter. We set input values of 0.1 and 0.01 for the *K*^trans^ and *v*_e_, respectively, and run the algorithm with 10,000 iterations and a tolerance of 10^−8^. The fitting procedure was also carried out with the user developed MATLAB function *fminsearchbnd* [27]. This function takes into consideration boundaries in the output values settings, which were set so as to consider only positive values. A comparison between the two functions’ results was done in terms of goodness of fit by estimating the Akaike Information Criteria (AIC) for each method using Eq. 7:

7 $$ AIC=2k+ nln\left(\frac{RSS}{n}\right)+\frac{2k\left(k+1\right)}{n-k-1} $$

where *n* is the number of data points, *k* the number of fitted parameters and RSS is the residual sum of squares [28].

**Fig. 1:**
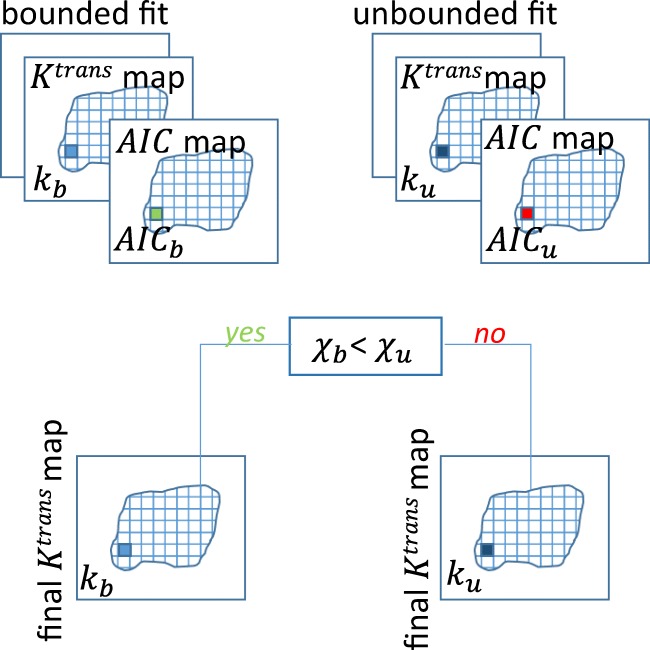
The fitting procedure for *K*^trans^. A bounded and unbounded fitting were calculated together with the Akaike Information Criteria (AIC) map (AIC_b_ and AIC_u_ for the bounded and unbounded procedure, respectively). The final value of *K*^trans^, for each voxel of the map, was the one obtained from the function with the lowest AIC (*k*_b_ when AIC_b_ < AIC_u_ and *k*_u_ vice versa). The same procedure was carried out for each parameter

In general, when performing model selection using the AIC, the model resulting with the lowest AIC value is the model that represents the best balance between complexity (i.e. the number of parameters) and goodness of fit (i.e. lower RSS). In this case, as the number of parameters is the same, only the goodness of fit is being tested. A comparison between the AIC maps relative to the bounded and unbounded procedure allowed the estimation of final *K*^trans^, *k*_ep_ and *v*_e_ maps where the value of each voxel was obtained from the fitting procedure with the best fit (lowest AIC) (Fig. 1).

#### Shutter speed model (SSM)

Both the TM and its extended version (2.3.3) embed the implicit assumption that equilibrium transcytolemmal water exchange (between the intracellular space and extracellular extravascular space) is infinitely fast, or that the system is in, what is called, the fast exchange limit [29]. Water exchange between the intracellular space and the extracellular extravascular space effects the degree of T_1_ shortening caused by CA. To account for this effect on the brain MRI signal amplitude, we applied the shutter speed model (SSM), which introduces a new pharmacokinetic parameter, the mean intracellular water molecule lifetime, τ_i_ [29].

In the SSM, Eq. 5 is applied to the distribution of the CA in the blood, without assuming that the equilibrium transcytolemmal water exchange kinetics are in the FXL. The longitudinal relaxation rate is measured as:

8 $$ {R}_{1b}(t)={r}_{1p}\left(1-h\right)\left[{CA}_p(t)\right]+{R}_{10p} $$

where *b* stands for the whole blood, *p* for the plasma and *h* the blood haematocrit. However, about half of the water in the blood is intracellular and cannot be accessed directly by the CA molecules [30]. The transport outside the erythrocytes therefore needs to be considered, as described by the two equilibria:

9 $$ {\mathrm{H}}_2{\mathrm{O}}_{\mathrm{i}}\leftrightarrow {\mathrm{H}}_2{\mathrm{O}}_{\mathrm{p}} $$

10 $$ {\mathrm{H}}_2{\mathrm{O}}_{\mathrm{p}}+{\mathrm{CA}}_{\mathrm{p}}\leftrightarrow {\mathrm{H}}_2\mathrm{O}\cdotp {\mathrm{CA}}_{\mathrm{p}} $$

The mean water molecule lifetime in commonly used CA is normally < 10^−7^ s, and the linear Eq. 5 is suitable for homogeneous solutions. In the case of erythrocytes, Eq. 10 is also considered fast for some commonly measured [CA_p_] values [30, 31]. After extravasation, the CA is commonly distributed into the interstitial extracellular space, at a rate defined by:

11 $$ {R}_1^{\ast }(t)={r}_{10}{p}_0\left[{CA}_0(t)\right]+{R}_{10} $$

where *R*_1_*(t) is the rate constant of the extravascular water signal, r_10_ is the interstitial CA relaxivity and *p*_0_ is the fraction of the extracellular tissue water. The application of Eq. 11 to biological tissues assumes that the interstitium is a homogeneous solution and that the system remains in the fast exchange limit. However, many studies have shown that, even though the equilibrium in Eq. 10 is fast, the FXL assumption is not true for all [CA_0_] values following a bolus injection [30]. [CA_0_] depends on the dimensions of the parenchymal cells that are generally much larger than erythrocytes and have a less water-permeable cytolemmae. Furthermore, tissue parenchyma cannot be considered as a single homogeneous solution and a single MRI voxel will constitute a number of compartments. The main result of this compartmentalization is given by:

12 $$ {R}_{1L}(t)=\left(1/2\right)\left\{2{R}_{1i}+{r}_{1o}\left[{CA}_o(t)\right]+\left({R}_{10}+{R}_{1i}+1/{\tau}_i\right)/{p}_o-{\left\{{\left(2/{\tau}_i-{r}_{1o}\left[{CA}_o(t)\right]-\left({R}_{10}+{R}_{1i}+1/{\tau}_i\right)/{p}_o\right)}^2+4\left(1-{p}_o\right)/{\tau}_i^2{p}_o\right]}^{\frac{1}{2}}\right\} $$

where *R*_1L_(t) is the long relaxation rate constant of the *shutter speed model*. *R*_1i_ is the H_2_O rate constant in the absence of exchange of CA and τ_i_ is the average intracellular lifetime of a water molecule. The SSM was fitted by substituting Eq. 12 in Eq. 6 using the MATLAB functions *fminsearch* and *fminsearchbnd*, similarly to the TM. The initial estimates for the SSM *K*^trans^ and *v*_e_ were taken as the outputs of the TM, while the initial estimate for τ_i_ was set at 0.1 [13]. The final *K*^trans^, *k*_ep_, *v*_e_ and τ_i_ maps were obtained from the fitting procedure with the best fit (lowest AIC value) as described in Fig. 1.

#### Extended Tofts model (ETM)

For highly perfused brain tissue, we applied the extended version of TM, which was introduced by Tofts in 1999 [32]. The ETMfits data with an additional parameter: the fractional plasma volume, *v*_p_ [32]. This model is able to distinguish the effects due to contrast leakage from those due to intravascular contrast. Eq. 6 becomes:

13 $$ \left[{CA}_o\right](T)={v}_p{C}_p(t)+{K}^{trans}{\int}_0^T{C}_p(t){e}^{-{K}^{trans}\left(T-t\right)/{v}_e} dt $$

The ETM was fitted by substituting Eq. 13 in Eq. 6 using the MATLAB functions *fminsearch* and *fminsearchbnd*, similarly to the TM and SSM. The initial estimates for ETM *K*^trans^ and *v*_e_ were again taken from the output of the TM and the initial estimate for *v*_p_ was set at 0.01 [13]. The final K^trans^, *k*_ep_, *v*_e_ and *v*_p_ maps were obtained from the fitting procedure with the best fit (lowest AIC) as described in Fig. 1.

#### Extended shutter speed model (ESSM)

The ESSM accounts for the contribution of the CA from the brain plasma compartment. This includes both *v*_b_, the fractional blood volume and *τ*_b_, the intravascular water molecule lifetime [13]. The contribution of the water signal comes from the three compartments (whole blood, EES and intracellular space) and is described by the matrix format of the Bloch equation [13]:

14 $$ \frac{d\boldsymbol{M}}{dt}=\boldsymbol{XM}+\boldsymbol{C} $$

where the column vectors are **M =** (M_b_, M_o_, M_i_) and **C** = (M_b0_R_1b_, M_o0_R_1o_, M_i0_R_1i_) with the ^1^H_2_O magnetization vector **M** ≈ to the signal **S**. The exchange matrix **X** is given by:

15 $$ \boldsymbol{X}=\left(\ \begin{array}{ccc}-\left({R}_{1\mathrm{b}}+{k}_{\mathrm{bo}}\right)\ & {k}_{\mathrm{ob}}& 0\\ {}{k}_{\mathrm{bo}}& -\left({R}_{1\mathrm{o}}+{k}_{\mathrm{ob}}+{k}_{\mathrm{oi}}\right)& {k}_{\mathrm{io}}\\ {}0& {k}_{\mathrm{oi}}& -\left({R}_{1\mathrm{i}}+{k}_{\mathrm{io}}\right)\end{array}\right) $$

The subscripts *b*, *o* and *i* stand for *blood*, *outside* space and *intracellular* space, respectively. *k*_bo_ (= 1/τ_b_) represents the blood to interstitium transfer of water; *k*_io_ (= 1/τ_i_) the transfer of water from the intracellular space to the interstitium; *k*_ob_ (proportional to 1/τ_o_) the EES to blood transfer and *k*_oi_ the EES to intracellular transfer [13]. For a SPGR sequence, the solution to Eq. 13 is the matrix form of the Ernst-Anderson relationship [33] which assumes that if the change in [CA] is relatively small during the acquisition, at every discrete data acquisition time point, the relaxation time can be estimated using:

16 $$ \boldsymbol{S}={\left[\mathbf{I}-{e}^{-\boldsymbol{X} TR}\left(\cos\ \alpha \right)\right]}^{-1}\left(\mathbf{I}-{e}^{-\boldsymbol{X} TR}\right){\boldsymbol{S}}_{\mathbf{0}}\left(\sin\ \alpha \right) $$

**I** is the identity matrix and **S**_**0**_ (= (S_b0_, S_o0_, S_i0_)) is the signal at baseline.

The ESSM was fitted by considering the measured signal E(t) as a combination of the signals in the three compartments (blood, outside space and intracellular space) [12] using:

17 $$ E(t)=\frac{S_b+{S}_o+{S}_i}{S_{b0}+{S}_{o0}+{S}_{i0}}-1 $$

Furthermore, Eq. 16 was simplified to:

18 $$ \boldsymbol{S}=\sin (a)\cdotp \boldsymbol{A}\cdotp {\boldsymbol{S}}_{\mathbf{0}} $$

where the column vectors are **S** = (S_b_, S_o_, S_i_) and $\boldsymbol{A}={\left[\mathbf{I}-{e}^{-\boldsymbol{X} TR}\left(\cos\ \alpha \right)\right]}^{-1}\left(\mathbf{I}-{e}^{-\boldsymbol{X} TR}\right)$.

The model was fitted by substituting Eq. 18 into Eq. 17 using the MATLAB function *fminsearch* function. The outputs of the SSM model were used as the initial estimates for *K*^trans^ and *v*_o_ and one third of the measured signal was used as the initial estimate for S_b0_, S_o0_ and S_i0_. Furthermore the initial estimates for *k*_bo_, *k*_ob_, *k*_oi_ and *k*_io_ were taken from literature defined values as 1.2, 1.5, 1.1 and 1.2, respectively [13]. The following parameters were also derived: τ_b_ = 1/k_bo_, τ_i_ = 1/k_io_, v_b_ = (k_ob_ − k_oi_ − ((k_io_/v_o_)f_w_) − k_io_)/((k_bo_ + k_io_)/v_o_) where *f*_w_ is tissue volume fraction accessible to mobile aqueous solutes (assumed to be a constant and set to 0.8) and *v*_i_ = 1 − (*v*_b_ + *v*_o_) [13].

#### No-effect model (NEM)

The no-effect model describes the case where [CA] within a brain voxel is so low that there is no permeability or vascular filling. In this situation, the MR signal is assumed to be unperturbed by the injection of the gadolinium-based CA and, as a consequence, the longitudinal relaxation rate does not change from its baseline value (R_10_). The system is therefore described by this value at all times such that the data is fitted by the constant R_10_.

### Arterial input function (AIF)

**Fig. 2:**
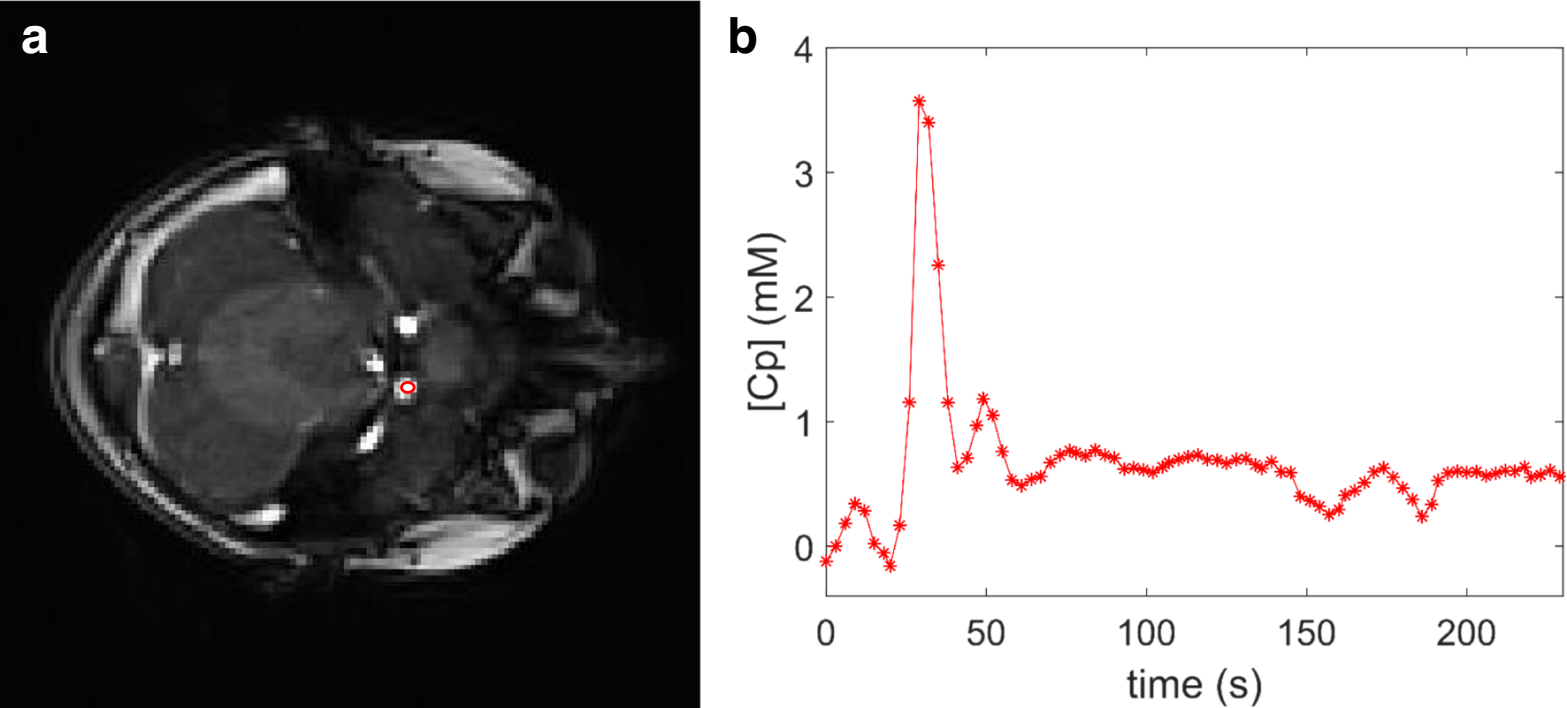
Measurement of the arterial input function (AIF). The VOI was placed in the carotid artery for the extraction of the AIF as shown in the axial T1 VIBE image in **a**. The time intensity curve for the concentration of contrast reagent in the plasma in the VOI indicated in red in **a** is shown in **B**

An additional ROI was drawn around the external carotid artery for the calculation of the image-derived AIF [34] (Fig. 2). Signal intensity curves were converted to R_1_-time curves by using the baseline signal intensity before the first pass of the CA as a reference, setting the haematocrit in the blood to 0.45, and getting the baseline blood *T*_1_ from the *T*_10_ map (Eq. 8).

### Model comparison

**Fig. 3:**
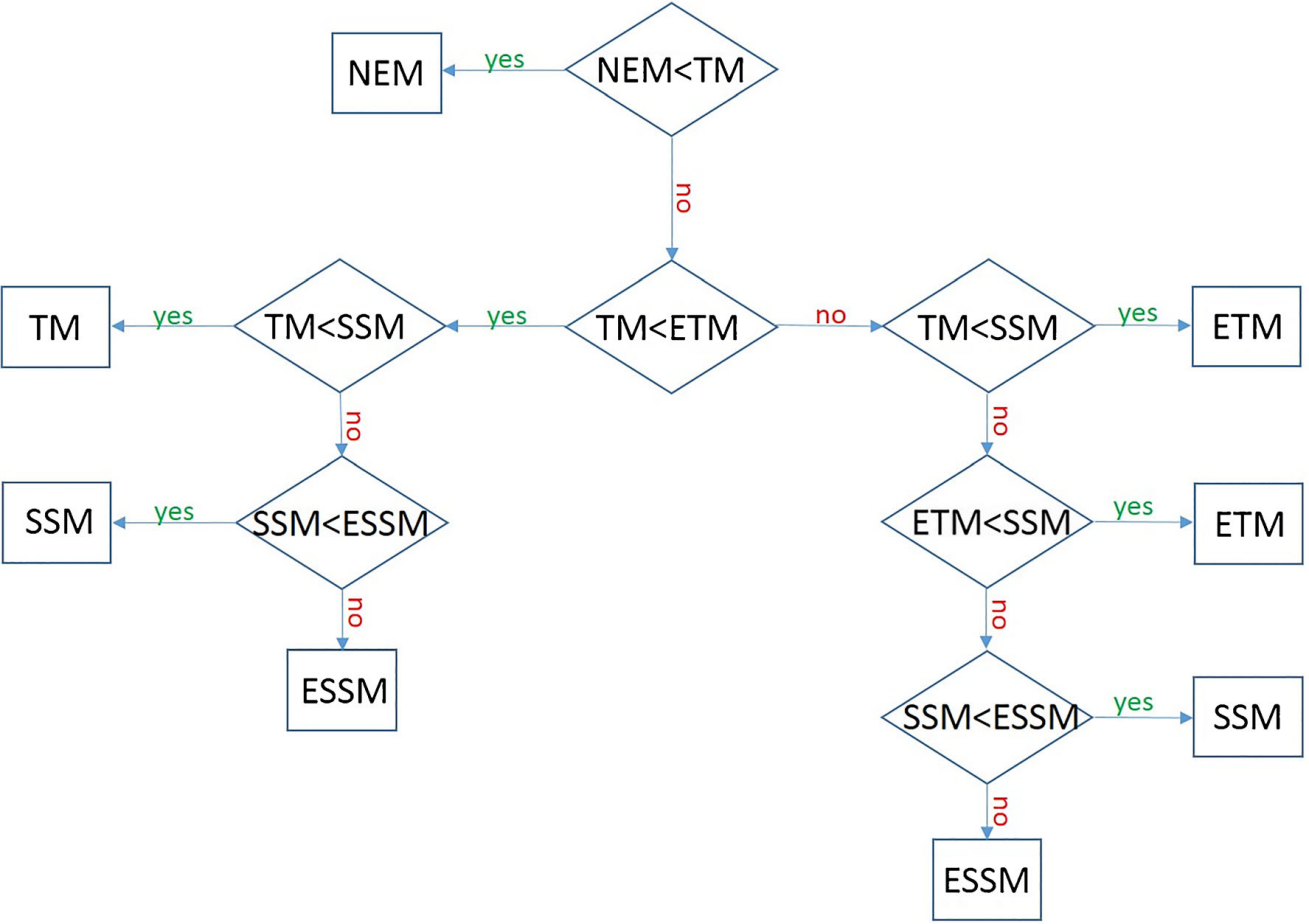
AIC model selection flowchart. The figure shows the hierarchical approach used to determine which model provided the best fit when using the Akaike Information Criteria (AIC)

A model comparison was carried out using the AIC to test for the best model in a given voxel. In particular, in the presence of exchange (where the NEM fails in the description of data), a voxel-wise comparison between models was carried out (with ETM and SSM being an extension of the TM, and the ESSM an extension of the SSM) to indicate which model provided the best fit using the AIC in each voxel. The selection method is shown in the flowchart in Fig. 3. The choice was expressed with a value of 1, 2, 3, 4 or 5 for the TM, ETM, SSM, ESSM and NEM, respectively, in a volumetric mask (same dimensions of the original tumour volume mask). The percentage of 1 s, 2 s, 3 s, 4 s and 5 s in each mask was evaluated to quantify the frequency of model choice. The model selection map was used to build final pharmacokinetic maps for the *K*^trans^, *k*_ep_ and v_e_, where, for each voxel, the value is the result of the fitting of the model of choice (lowest AIC).

#### Stability of pharmacokinetic parameters

Once the model selection had been selected, the stability of each parameter within the selected models was evaluated in a simulation environment. Tissue curves were generated back from the extracted pharmacokinetic parameters and signal intensity curves were calculated with the inverse formula of Eq. 4. White Gaussian noise was added to the signal intensity curves using a signal to noise ratio (SNR) of 20. The SNR value for the simulated data was set by evaluating the SNR of the acquired data from the second and third phase of the dynamic acquisition sequence using the subtraction method [35]. The simulated noisy signal intensity curves were reconverted to noisy tissue concentration curves, and fitted to the selected pharmacokinetic model. This procedure was repeated 500 times for every kinetic parameter and the variability of each parameter was expressed in terms of coefficient of variation (CV): the percentage ratio between the standard deviation and the mean.

## Results

### Model selection and parameter variability

**Fig. 4:**
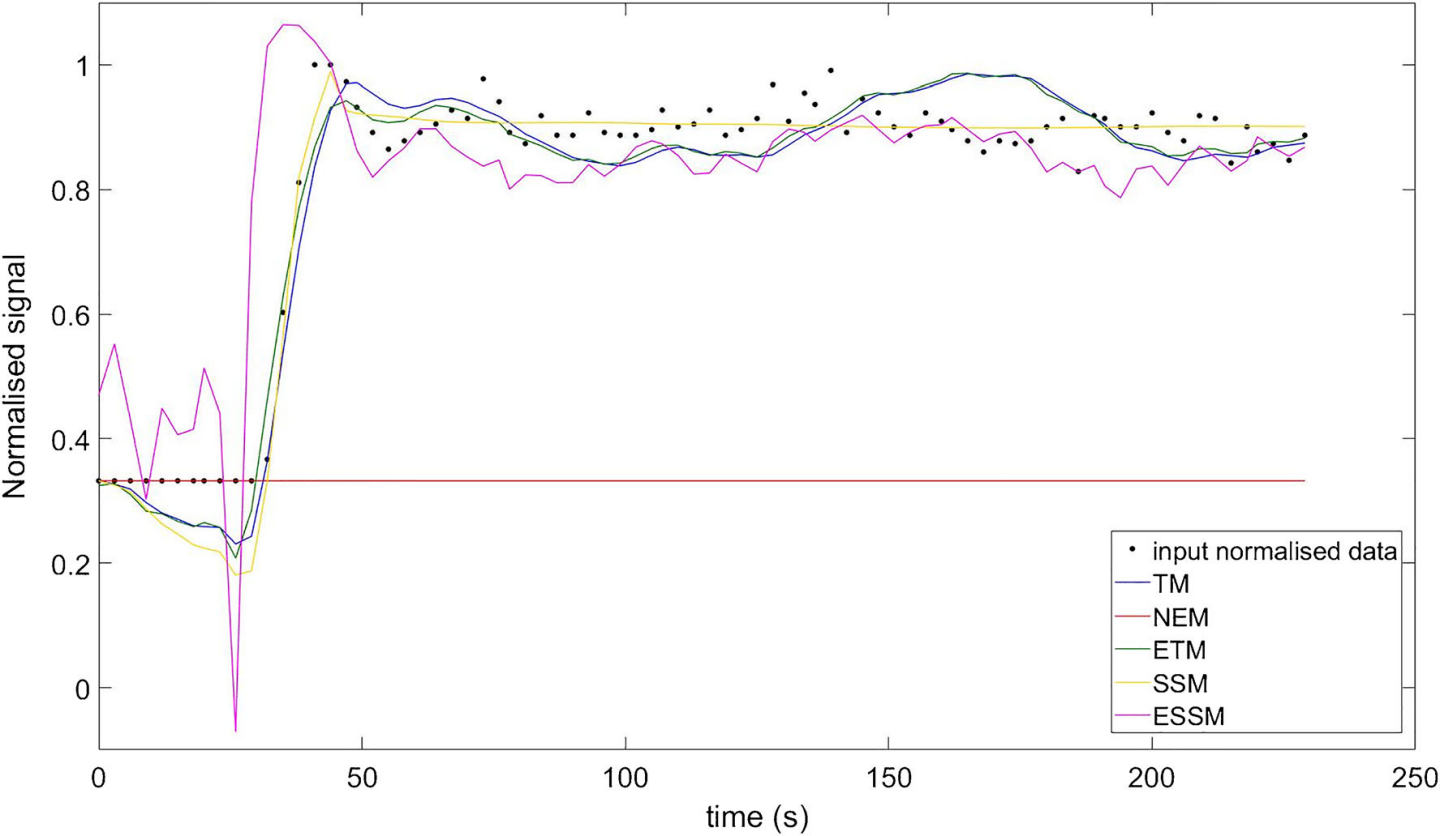
Normalized signal intensity curves in a voxel of an enhancing lesion fitted with the NEM (red), TM (blue), ETM (green), SSM (yellow) and ESSM (pink). The quality of fitting was evaluated with the Akaike Information Criteria. AIC value: − 103 for the NEM, − 445 for TM, − 454 for ETM, − 531 for SSM and − 291 for ESSM

**Fig. 5:**
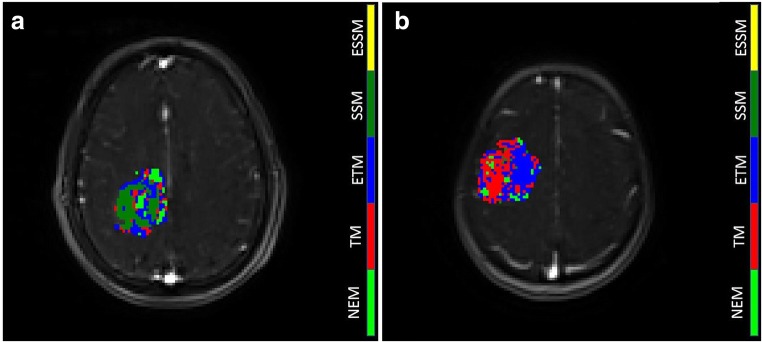
Statistical model comparison for two lesions. Each colour is representative of the model which best fitted the input data. An example of one slice of an enhancing (**a**, WHO grade IV) and non-enhancing (**b**, WHO grade II) lesion is shown

**Fig. 6:**
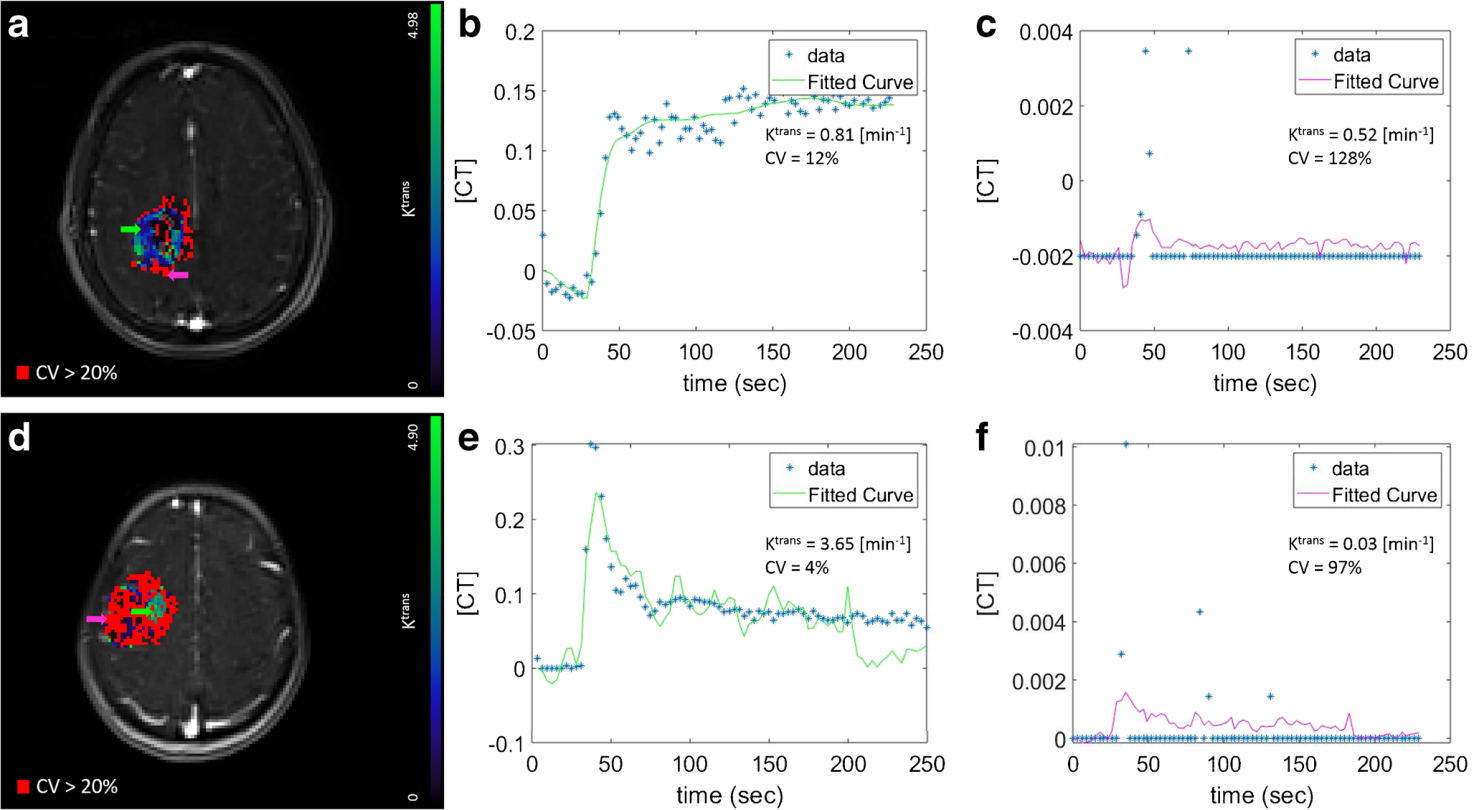
The stability of each pharmacokinetic parameter extracted from the fitting of the model of choice was evaluated, for each lesion, in terms of coefficient of variation in a simulation environment. **a** and **d** show two *K*^trans^ maps in enhancing (**a**) and non-enhancing (**d**) lesions. The reliability of DCE-MRI data was evaluated by setting a threshold of 20% for the CV. This is overlaid on the *K*^trans^ maps in **a** and **d**, shown in red, such that only values of *K*^trans^ under this threshold are displayed on the blue/green colour map. Two tissue activity curves (TACs) relative to two reliable (CV = 12% and CV = 4%) voxels are plotted in **b** and **e**. **c** and **f** show the TACs relative to two unreliable voxels (CV = 128% and CV = 97%)

The behaviour of each model was assessed by studying the quality of fit for each of the models. The input data, together with the fitted curves were normalized by the maximum value of the input data in order to compare results from the different fits. An example of a comparison of fit is shown in Fig. 4. For each tumour, a map with the result of the statistical comparison among models was built (Fig. 5). In this map, each colour represents the model for which the voxel-wise AIC value was lowest: The selected model of choice representing majority of low AIC was NEM (35.5% of voxels), followed by the ETM (32%), TM (28.2%), SSM (4.3%) and ESSM (< 0.1%). Fig. 5 shows the model selection map evaluated for two different lesions. Furthermore, final pharmacokinetic maps, for the *K*^trans^, *k*_ep_ and *v*_e_, were built considering the model selection procedure. Within the final pharmacokinetic maps, each voxel was represented by the model with the best fit (lowest AIC) within that voxel. For each of these maps, the stability of each parameter and for each lesion was presented in terms of CV maps. Table 1 shows the results of the stability test on the final *K*^trans^ map. The total number of voxel of the lesion, for each patient, together with the average *K*^trans^ value evaluated over the whole tumour, is shown. Furthermore, the map was thresholded with a CV lower than 10, 20 and 50%, and the resultant percentage of preserved voxels (and their average value) is shown. Fig. 6 shows two final *K*^trans^ maps with their CV map overlaid on them (A and D).

**Table 1:** K^trans^ stability test results

					CV ≤ 10%		CV ≤ 20%		CV ≤ 50%	
	Diagnosis	WHO grade	TOT n. voxel	Whole tumour *K*^trans^ mean	% n. voxel	*K*^trans^ mean	% n. voxel	*K*^trans^mean	% n. voxel	*K*^trans^mean
P01	Dysembryoplastic neuroepithelial tumour	I	791	0.19	18	0.57	36	0.32	51	0.28
P02	Astrocytoma	II	3284	0.88	3	1.81	15	1.09	48	1.10
P03	Oligodendroglioma	II	1266	0.83	9	1.30	20	1.22	38	1.43
P04	Oligodendroglioma	II	2309	1.69	8	1.32	14	1.16	39	2.49
P05	Astrocytoma	II	4321	0.72	13	1.62	22	1.67	45	1.32
P06	Astrocytoma	II	2809	0.70	5	1.31	18	1.15	58	1.01
P07	Astrocytoma	II	2904	0.48	8	2.06	16	1.49	33	1.03
P08	Oligodendroglioma	III	1762	0.56	9	2.31	24	1.38	51	0.90
P09	Oligodendroglioma	III	3690	1.47	7	2.12	29	2.55	67	1.96
P10	Astrocytoma	III	8887	0.69	7	2.52	16	1.99	46	1.09
P11	Astrocytoma	III	1899	0.46	7	1.76	15	1.28	32	0.96
P12	Glioblastoma	IV	2704	0.25	13	0.42	22	0.50	38	0.47
P13	Glioblastoma	IV	6803	0.41	35	0.81	48	0.69	64	0.57
P14	Glioblastoma	IV	1958	1.46	46	1.31	64	1.25	81	1.41

The stability test consisted in the evaluation of maps of the coefficient of variation (CV). The table shows the stability of the final *K*^trans^ maps. For each patient, the first two columns show the total number of voxels of the lesion and the average value of *K*^trans^ evaluated across the whole lesion. Following, the percentage of voxels with a CV lower than 10, 20 and 50% and the relative mean *K*^trans^ values are shown. The WHO grade and diagnosis of each lesion are also reported

## Discussion

In this study, we showed that in over one third of the brain tumours voxels (35.5%), standard model fitting of DCE-MRI data was inconclusive and therefore fitting these models to the data would lead to incorrect perfusion parameters. Considering a CV of 20%, only ≈ 25% of remaining voxels were found to be reliable. The reproducibility of this technique and, as a consequence, its reliability can be improved by improving the main sources of variability in quantitative DCE-MRI (the acquisition method and the quantification process) [16]. Nonetheless, in most studies, the intrinsic heterogeneity of the lesion is ignored by quantifying the perfusion with one single pharmacokinetic model and, more importantly, carrying out statistical analyses on one single whole tumour statistic (usually the average). In this study, we investigated the reliability of DCE-MRI focusing on the quantification analysis. We took into consideration the particularly heterogeneous nature of brain tumour vascular permeability due to the presence of the BBB, as well as existence of necrotic regions and provided a method to identify robust DCE-MRI data based on a model selection procedure and a stability test.

### DCE-MRI models

MR scanners usually employ post processing perfusion tools which fit DCE data with the TM. This model (together with its extended version [32]) considers the system in a fast exchange limit [36, 37], assuming an infinitely fast transcytolemmal water exchange between the EES and the intracellular space, which does not affect the overall signal decrease [36, 37]. Therefore, many studies on the cell membrane water permeability coefficient have shown FXL to be physiologically unreasonable and inconsistent [38]. The shutter speed model was introduced to reflect a more realistic tissue environment. The model accounts for the intercompartmental water exchange effect, modelling this non-infinitely-fast exchange with the mean intracellular water molecule lifetime τ_i_. In 2005, Li et al. introduced a second generation of the shutter speed model which considers also a non-infinitely fast equilibrium transendothelial water exchange.

### Model comparison and stability

The heterogeneity that exists in brain tumours means that one model is insufficient in explaining the different biologies that exist in different tumour regions. Multiple pharmacokinetic models are required for a complete description of the tissue. This variability is testified by the model selection procedure which showed how, in a single slice of one tumour, multiple models perform better. This result confirms the study of Bagher-Ebadian where they implemented a selection method based on nested models [3]. They found that in the necrotic core of the tumour, models describing vascular filling with no microvascular leakage (similar to the TM) and leakage without vascular reabsorption were selected because of the lack of blood flow. They also hypothesised that the model describing leakage with reabsorption (similar to the ETM) would be selected in the fast growing rims of the lesion. Our results show that there are a number of regions in the tumour where the CA exudation is prevented by the BBB and where the concentration of CA is so low that the evidence of perfusion is missing. In this case, the use of the NEM is recommended as the use of different models could result only in overfitting the data. In fact, our results showed that no leakage of the CA into the interstitium and the lack of flow of the CA through the tissue made the NEM the model of choice for the majority of regions, particularly in the non-enhancing lesions (37.5% of voxels). The result is very close to the ETM (32%), which was the model of choice in the enhancing lesions (54.8%). This suggests that, in areas where there is enhancement, a model with three parameters performs better and that the choice is dependent on the underlying state of the tissue. In fact, both the ETM and SSM are fitted by three parameters but the third parameter is very different between the two models (*v*_p_ for the ETM describing a vascular component in the tissue, and τ_i_ for the SSM describing the transcytolemmal water exchange). Furthermore, with the implementation of the ESSM, we saw that the transendothelial water exchange did not have any impact on the signal (compared with parameters derived by the fitting of simpler models). It is necessary to consider that the ESSM required nine parameters to be fitted and that the cost of fitting extra parameters is often contrary to the principle of parsimony. In fact, in fitting data to a noise-limited dataset, the estimation could be very poor and dependent on the optimization procedure itself (the initial conditions, for example) [3]. We compared the AIC values from the different fitting procedures to check whether a model with more parameters is more appropriate than a simple one. The ESSM was selected as the model of choice by < 0.1% of voxels, indicating that a model with three parameters performed better in the description of brain tumours and further confirming the poor quality of fit observed for the ESSM model. Our outcome agreed with the results of Duan et al. [39]. Using representative in silico and clinical (cervical cancer) DCE-MRI data, they demonstrated the sensitivity of complicated models (parameters > 3) to noise and their decreasing probability of being selected in low signal-to-noise data [39].

The reliability of DCE-MRI data is not only based on the goodness of fit of the chosen pharmacokinetic model, but also on the robustness of the extracted parameters. For this reason, we assessed, for each lesion and for each parameter, the coefficient of variation. We worked in a simulation environment where we added Gaussian noise to our signal and we fitted the noisy curves 500 times. This procedure resulted with a heterogeneous distribution of CVs that was not linked to contrast enhancement. In fact, Fig. 6 shows the plot of four different tissue activity curves together with the *K*^trans^ value and its CV in an enhancing and non-enhancing tumour. The curves in Fig. 6b, c belong to the same enhancing lesion and while they correspond to similar *K*^trans^ values of 0.81 and 0.52 [1/min], they varied by 12 and 128%, respectively. On the other hand, the curves in Fig. 6e, f belong to the same non-enhancing lesion and show regions with both a low (4%) and a high (97%) variability.

We set three different thresholds for the CV to evaluate the variability of the *K*^trans^. Table 1 shows the percentage of voxels and their relative mean *K*^trans^ value at different CVs thresholds (10, 20 and 50%), for each lesion and for each patient. Higher grade glioma tend to have more voxels with a lower CV and also a more stable value of *K*^trans^ while, for some of the other patients, the mean value of *K*^trans^ is highly affected by the portion of voxels taken into consideration (P04, P07). This result confirmed the improper use of one average value in statistical comparisons of brain tumours. Not only because of the heterogeneity of the tissue under investigation but also, and more importantly, because it is affected by the reliability of fit within voxels.

Finally, Fig. 6 gives a graphical representation of this effect showing *K*^trans^ values under the 20% threshold of CV covering only 25% of voxels (an average percentage value evaluated among all patients). This result suggested that only this selection of voxels represents robust values, which can be used in the following statistical analyses, as, more importantly, in clinical evaluations. The selection of the threshold that makes DCE-MRI robust is, however, dependent on the effect size that is being measured and hence will vary across studies.

The main limitation of this study is the small size of the dataset. Furthermore, the sensitivity of DCE-MRI data to water exchange effect was reduced by the 26° flip angle acquisition (exchange-minimized approach) [40]. As a consequence, the precision of the τ_i_ parameter extracted might be low.

In conclusion, DCE-MRI methods hold great promise for quantitative in vivo evaluation of permeability and vascular properties under different pathophysiological conditions. It allows us to identify, and quantitatively measure, smaller changes in permeability for pathological conditions effecting the BBB, than would be observed through visuassessment of post-contrast T_1_-weighted images. Different models yield different pharmacokinetic parameters and, for this reason, a model selection is critical for the appropriate analysis of DCE-MRI time courses based on the regional tissue biology, specifically permeability and vasculature. Future work needs to assess the physiological basis for each model in the reliable selection of DCE-MRI data. The applicability of each model depends on the physiology, anatomy and heterogeneity of the tumour and the tumour microenvironment. In addition, due to the noisy nature of DCE-MRI data, a model selection procedure alone is not enough: pharmacokinetic parameters need to be validated with a stability test in order to give only robust results for statistical analyses and clinical evaluation.
